# Lower risk of smoking-related cancer in individuals with familial hypercholesterolemia compared with controls: a prospective matched cohort study

**DOI:** 10.1038/s41598-019-55682-x

**Published:** 2019-12-17

**Authors:** Henriette W. Krogh, Karianne Svendsen, Jannicke Igland, Liv J. Mundal, Kirsten B. Holven, Martin P. Bogsrud, Trond P. Leren, Kjetil Retterstøl

**Affiliations:** 1 Department of Nutrition, Institute of Basic Medical Sciences, University of Oslo, Oslo, Norway; 2 The Lipid Clinic, Department of Endocrinology, Morbid Obesity and Preventive Medicine, Oslo University Hospital, Oslo, Norway; 3 Department of Global Public Health and Primary Care, University of Bergen, Bergen, Norway; 4 National Advisory Unit on Familial Hypercholesterolemia, Department of Endocrinology, Morbid Obesity and Preventive Medicine, Oslo University Hospital, Oslo, Norway; 5 Unit for Cardiac and Cardiovascular Genetics, Oslo University Hospital, Oslo, Norway; 6 Department of Health and Social Sciences, Institute of Health and caring Science, Western Norway University of Applied Sciences, Bergen, Norway

**Keywords:** Cancer epidemiology, Cardiovascular genetics, Risk factors, Lipids, Lipoproteins

## Abstract

According to guidelines, individuals with familial hypercholesterolemia (FH) shall receive lifestyle intervention and intensive lipid-lowering treatment from early in life to reduce the risk of coronary heart disease. Our aim was to study if treatment of FH also could affect risk of lifestyle-related cancer. We presented cumulative incidence of total cancer and lifestyle-related cancer sites in individuals with genetically verified FH (n = 5531) compared with age and sex matched controls (n = 108354). Individuals with FH had 20% lower risk of smoking-related cancer compared with the control population [HR 0.80 (95% CI, 0.65–0.98)], in particular men with FH at 40–69 years at age of diagnosis with HR 0.69 (95% CI, 0.49–0.97). The FH population and controls had similar rates of total cancer [HR 0.97 (95% CI, 0.86–1.09)], cancer related to poor diet [HR 0.82 (95% CI, 0.59–1.15)], cancer related to physical inactivity [HR 0.93 (95% CI, 0.73–1.18)], alcohol-related cancer [HR 0.98 (95% CI, 0.80–1.22)] and cancer related to obesity [HR 1.03 (95% CI, 0.89–1.21)]. In summary, we found reduced risk of smoking-related cancer in individuals with FH, most likely due to a lower prevalence of smoking. Implications of these findings can be increased motivation and thus compliance to treatment of hypercholesterolemia.

## Introduction

A combination of lifestyle intervention including dietary advice, physical activity, smoking cessation and lipid-lowering medication, usually statins, is vital in the treatment of hypercholesterolemia^1^. Familial hypercholesterolemia (FH) is a disorder usually caused by a mutation in the low density lipoprotein (LDL) receptor gene, causing elevated plasma levels of LDL cholesterol (LDL-C) which leads to increased risk of coronary heart disease (CHD)^2^. Individuals with FH are therefore recommended lifelong lifestyle intervention and lipid-lowering medication from the age of 8–10 years or from the time of diagnosis^3^. Consequently, it has been demonstrated that both children and adults with FH have a healthier lifestyle than controls^4,5^. Cardiovascular disease (CVD) and cancer are the two main types of non-communicable diseases (NCDs). These diseases share several risk factors such as tobacco use, an unhealthy diet including low fruit and vegetable intake, lack of physical activity, and alcohol use^6^. Accordingly, lifestyle advice to prevent CVD and cancer overlap^6^. In the present study, we therefore investigated whether treatment for FH results in lower risk of total and lifestyle-related cancer in people with FH compared with an age and sex matched control population.

## Materials and Methods

### Study design and population

**Figure 1:**
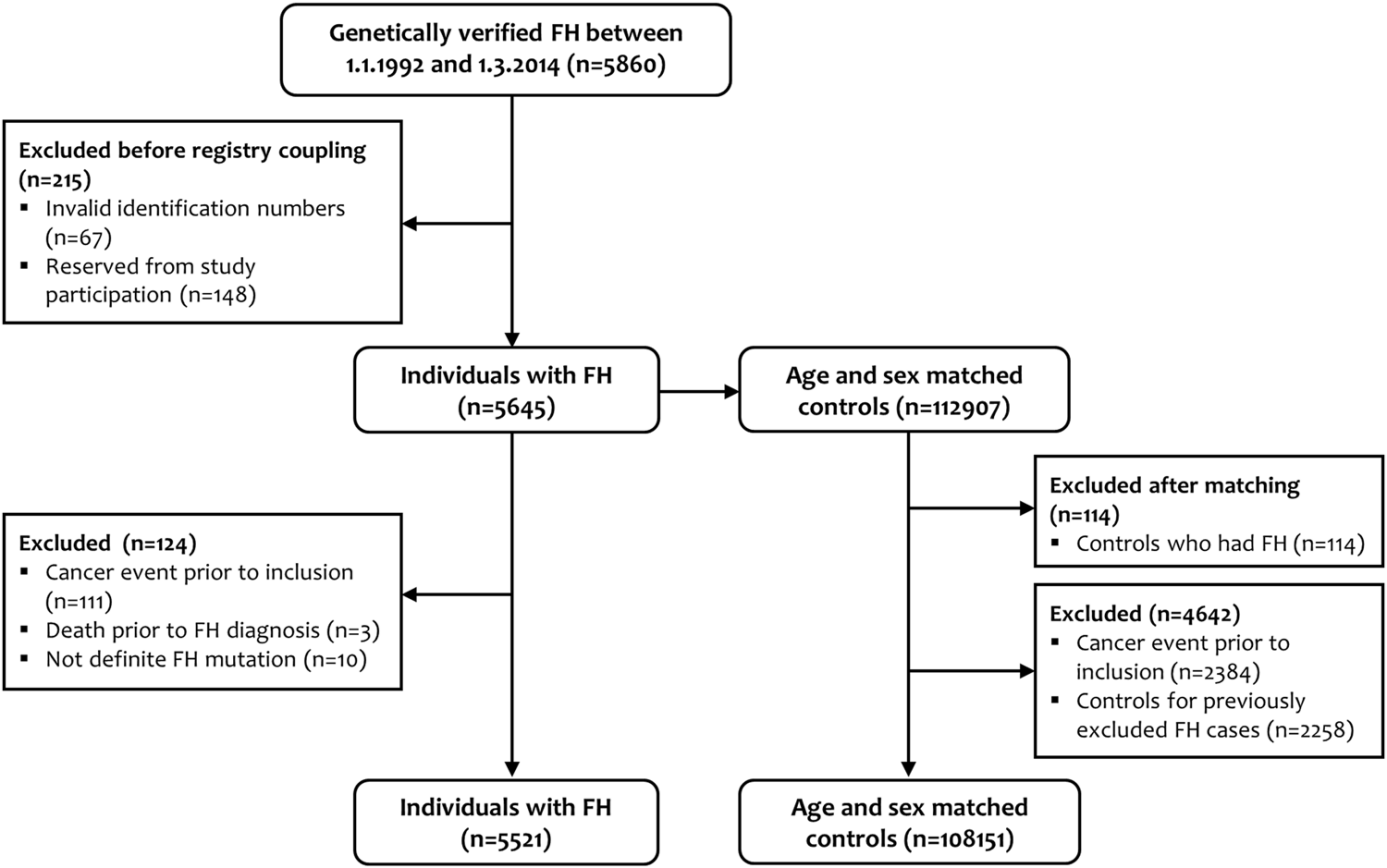
Flow chart of individuals with familial hypercholesterolemia (FH) and age and sex matched controls included in the study.

This study is a prospective matched cohort study comprising of a sample of individuals with genetically verified FH, and age and sex matched controls (1:20). The FH sample was obtained from the Unit for Cardiac and Cardiovascular Genetics (UCCG) Registry for individuals with genotyped FH, at Oslo University Hospital. The UCCG Registry has been described elsewhere^7,8^. In brief, since its establishment in 1992 all individuals with genetically verified FH are asked for consent to be included in the registry. The UCCG Registry currently holds 8511 individuals with genetically verified FH^9^. In the present study, birth year, sex, and date and age at inclusion to the registry were extracted from the UCCG Registry. Inclusion date to the registry was equal to date of genetic diagnosis after 1.1.1992. In total 744 individuals were missing exact inclusion date due to diagnosis prior to 1992 and were assigned inclusion date 1.1.1992. In this study we included 5645 individuals with FH who were registered between 1.1.1992 and 1.3.2014 (deadline of study reservation). The FH population was matched on age and sex on inclusion date to the UCCG Registry, to 112907 controls randomly drawn from the Population Registry of Norway. After excluding subjects for various reasons, as shown in Fig. 1, we analysed a cohort of 5521 individuals with FH and 108151 age and sex matched controls for their first cancer event or death from cancer in the period from baseline (date of inclusion to the registry) and until latest 31.12.2017 (last day of registry coupling) (Fig. 1).

This study was approved by The Regional Committee of Medical and Health Research Ethics for South-Eastern Norway (reference 2011/1343 REK Sør-Øst B), by the Norwegian Data Protection Official at Oslo University Hospital, and complies with the Declaration of Helsinki. Due to the nature of this study, we were granted exemption from the obligation to gain informed consent from the FH population and controls. However, the individuals with FH were informed about the study by a letter of information and were given the opportunity to reject having their information linked to the registries, as previously described^7,10^.

### Cancer outcomes

The FH population and controls were linked (by means of the 11-digit personal identification number unique to each Norwegian resident) to the Cancer Registry of Norway (CRN) and the Norwegian Cause of Death Registry (NCoDR) in order to obtain information on cancer incidence and mortality (underlying cause of death in the death certificates). Endpoints were defined according to the International Classification of Diseases (ICD), version 10 (ICD-10) and for NCoDR also ICD-9 (1992–1995) codes. All ICD-9 codes were converted to the ICD-10 codes that are shown in Table 1.

**Table 1:** Definition and categorization of total and lifestyle-related cancer sites, number of cases and hazard ratios for FH women and men combined compared with the matched control population.

Cancer types grouped by risk factors	Description	ICD-10 codes^a^	FH (n)	Controls (n)	HR (95%CI)
Total cancer	All cancers	C00-C99	289	5579	0.97 (0.86–1.09)
Smoking-related cancer*	Cancer types known to be associated with smoking, including mouth, pharynx, larynx, lung, esophagus, stomach, liver, pancreas, colorectum, kidney, renal pelvis, bladder, uterine cervix, and acute myeloid leukemia	C00-C16, C18-C22, C25, C26.0, C30-C34, C53, C64-C66, C67, C92.0	97	2220	0.80 (0.65–0.98)
Cancer related to poor diet^b,**^	Cancer types known to be associated with a poor diet, including colorectal and stomach	C16, C18-C21	36	801	0.82 (0.60–1.15)
Cancer related to physical inactivity**	Cancer types known to be associated with lack of physical activity, including colorectum^c^, breast, and endometrium	C18-C19, C26.0, C50, C54-C55	72	1455	0.93 (0.73–1.18)
Alcohol-related cancer**	Cancer types known to be associated with alcohol intake, including mouth, pharynx, larynx, esophagus^d^, stomach, liver, colorectum, and breast	C00-C15, C18-C22, C26.0, C32, C50	90	1718	0.98 (0.80–1.22)
Cancer related to body fatness and weight gain**	Cancer types known to be associated with excess body weight, including mouth, pharynx, larynx, esophagus^e^, stomach^f^, liver, gallbladder, pancreas, colorectum, kidney, breast, endometrium, ovary, and prostate^g^	C00-C15, C16.0, C18-C25, C26.0, C32, C50, C54-C56, C61, C64	172	3131	1.03 (0.89–1.21)

*Source: Cancer Facts & Figures 2018, American Society of Cancer. **Source: The 2018 Third Expert Report, World Cancer Research Fund/American Institute for Cancer Research. ^a^Origin of the tumor. Converted based on localization codes and morphology codes, and designated as the ICD-10 group (or converted from ICD-9), ^b^Poor diet = Red and processed meat consumption, low dietary fiber and wholegrain consumption, and low dietary calcium consumption, ^c^Colon only, ^d^Squamous cell carcinoma only, ^e^Adenoncarcinoma, ^f^Cardia only, ^g^Advanced prostate cancer. FH, familial hypercholesterolemia; n, number of cases; HR, hazard ratio, CI, confidence interval.

As stated earlier, CVD and cancer have several lifestyle-related risk factors in common^6^. We identified and categorized cancer types according to World Cancer Research Fund (WCRF) & American Institute for Cancer Research (AIRC) and Cancer Facts & Figures from the American Cancer Society (ACS) who have defined groups of lifestyle-related cancers^11,12^. Risk factors that according to WCRF&AICR and ACS have sufficient or strong (either convincing or probable) evidence for causing cancer in humans were included in the study^11,12^. Hence, the lifestyle-related cancer sites included smoking-related cancer^11^, cancer related to poor diet [red and processed meat consumption, low dietary fiber and wholegrain consumption, and low dietary calcium consumption], cancer related to physical inactivity, alcohol-related cancer, and cancer related to body fatness and weight gain^12^, as shown with corresponding ICD-10 codes in Table 1.

### Statistical analysis

Baseline for start of follow-up was defined as date of the pathogenic genetic diagnosis for individuals with FH, and the same date was chosen for each of the FH patients’ corresponding controls. Follow-up time was calculated as time from baseline until date of cancer diagnosis or death (from CRN or NCoDR), or 31.12.2017, whichever came first. Cumulative incidence curves for risk of cancer with death treated as competing event were constructed separately for the FH population and the control population using the stcompet-package in Stata. The curves are presented graphically with age as the time scale. Risk of cancer among individuals with FH was compared with age and sex matched controls in terms of hazard ratios (HR) with 95% confidence intervals (95% CI) obtained from Cox proportional hazards regression, stratified on matched risk set to take the matching into account. Because of the already known increased risk of CVD in the FH population^7^, we also performed competing risk regression with deaths treated as competing event in order to take into account that individuals with FH might die from CVD before they get cancer. The results were however very similar (only a small change in the second decimal of HRs) and we therefore only present results from Cox regression. Because lifestyle advice may affect women and men differently, and because age at time of FH diagnosis also may modify the risk of cancer, we performed analyses stratified on sex and age groups (0–39, 40–69 and age 70+). All analyses were performed with Stata version 15 and 5% was used as the significance level for all statistical tests.

## Results

Incidence of total cancer and grouped lifestyle-related cancers for the FH population and the control population are summarized in Table 1. Baseline characteristics of the study populations are shown in Table 2. Age at baseline corresponds to age at genetic FH diagnosis for the majority of the sample and was 33.7 ± 18.9 in the FH population (n = 5521) and 33.2 ± 18.7 in the age and sex matched controls (n = 108151) (Table 2).

**Table 2:** Baseline characteristics of the study population.

	Total	Control	FH
n	113672	108151	5521
Sex, n (%)			
Women	58397 (51.4)	55547 (51.4)	2850 (51.6)
Men	55275 (48.6)	52604 (48.6)	2671 (48.4)
Age, mean (sd)*	33.2 (18.7)	33.2 (18.7)	33.7 (18.9)

*Age at FH diagnosis. FH, familial hypercholesterolemia; n, number of cases; sd, standard deviation.

### Total cancer

**Figure 2:**
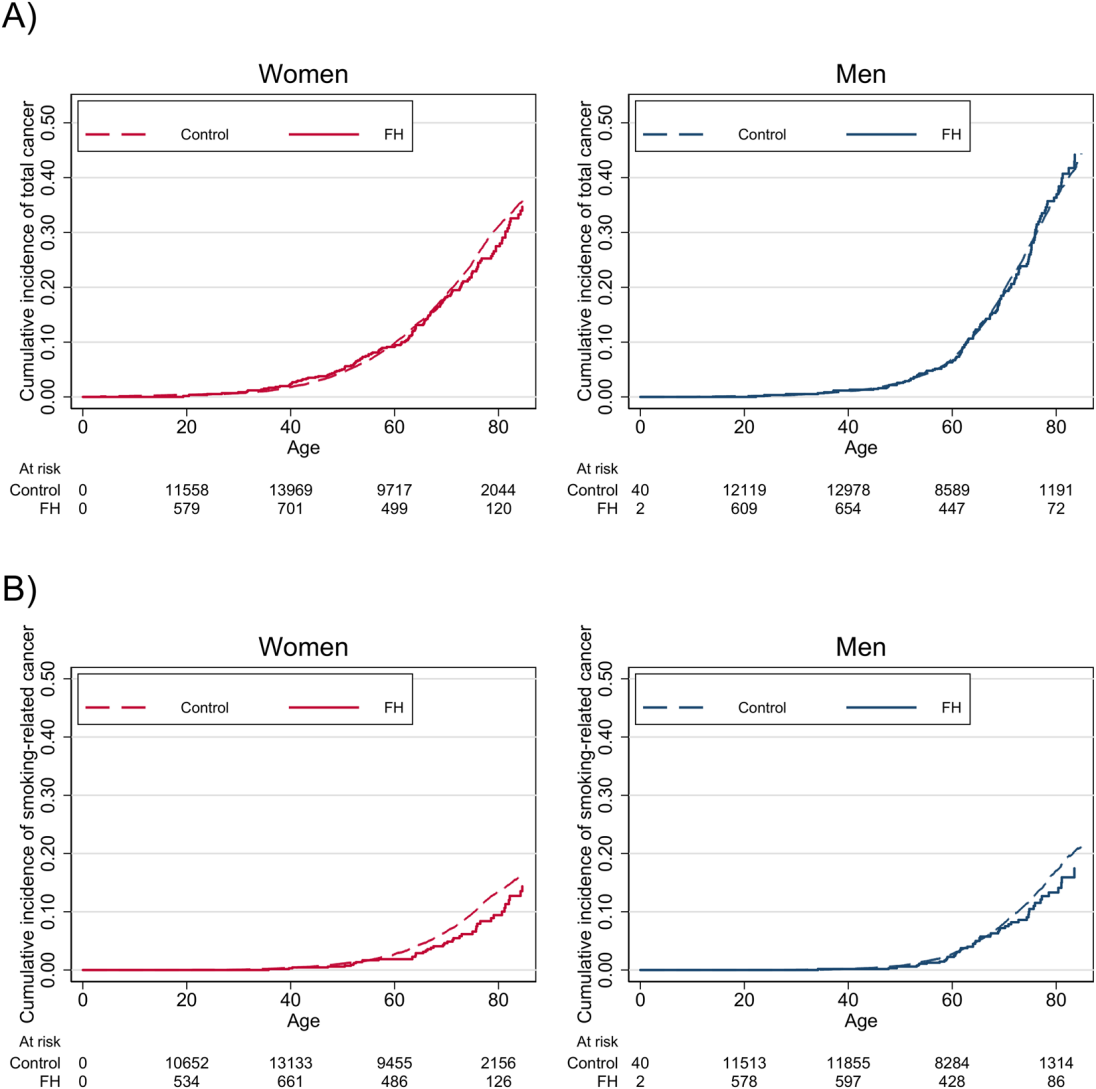
(**A**,**B**) Cumulative incidence curve for total cancer (**A**) and smoking-related cancer (**B**) in individuals with familial hypercholesterolemia (FH) compared with matched controls, stratified on sex and age (inclusion to the UCCG Registry) as the time scale.

A total of 289 cancer cases were observed in the FH population and 5579 cases in the control population (Table 1**)**. In the FH population, the total number of person years of follow-up was 71253 and the median time to cancer diagnosis among those who developed cancer was 8.7 years. In the control population, the corresponding numbers were 1366647 person years and 8.8 years, respectively. As illustrated in Fig. 2A, the cumulative incidence curve for total cancer increased with age, similarly for the FH population and controls. The total incidence per thousand person years of follow-up was 3.99 (95% CI, 3.88–4.09) in the control population and 4.06 (95% CI, 3.61–4.55) in the FH population. The HR for incidence of total cancer for the FH population versus the control population was 0.97 (95% CI, 0.86–1.09) (Table 1). Similarly, there was no significant difference in total cancer between the FH population and controls stratified by men [HR 0.97 (95% CI, 0.82–1.15)] and women [HR 0.98 (95% CI, 0.83–1.15)], nor for any of the three age groups (0–39, 40–69 and age 70 + at baseline) (Table 3).

**Table 3:** Cases of total cancer and hazard ratios for the FH population compared with the control population, with stratification on age at baseline.

Total study population				Women		Men	
	Cases	HR (95% CI)*	p-value	Cases	HR (95% CI)*	Cases	HR (95% CI)*
Age 0–39							
Control	923	1		589	1	334	1
FH	50	1.09 (0.82–1.44)	0.57	28	0.95 (0.65–1.39)	22	1.33 (0.87–2.05)
Age 40–69							
Control	4050	1		1962	1	2088	1
FH	203	0.95 (0.82–1.09)	0.44	100	0.98 (0.80–1.20)	103	0.91 (0.75–1.11)
Age 70+							
Control	606	1		337	1	269	1
FH	36	0.98 (0.70–1.37)	0.90	20	0.99 (0.63–1.57)	16	0.96 (0.58–1.60)

*HR from Cox proportional hazards regression stratified on matched case set. FH, familial hypercholesterolemia; HR, hazard ratio; CI, confidence interval.

### Lifestyle-related cancer

In the FH population, we observed 97 cases of smoking-related cancer, whereas 2220 cases were observed in the control population. Individuals with FH had 20% lower risk of smoking-related cancer compared with the control population [HR 0.80 (95% CI, 0.65–0.98)]. In the age group 40–69 years at baseline, the FH population was 27% less likely to have smoking-related cancer compared with controls, as demonstrated with a HR of 0.73 (95% CI, 0.57–0.94). The strongest relative reduced risk was observed for men with FH aged 40–69 years at baseline, where an incidence rate per thousand person years of follow-up for smoking-related cancer was 3.94 (95% CI, 3.69–4.20) compared with 2.85 (95% CI, 2.04–3.99) for controls [HR 0.69 (95% CI, 0.49–0.97]. The corresponding HR for women aged 40–69 years was 0.79 (95% CI, 0.56–1.13) (Table 4 and Fig. 2B).

**Table 4:** Cases of smoking-related cancer and hazard ratios for the FH population compared with the control population, with stratification on age at baseline.

Total study population				Women		Men	
	Cases	HR (95% CI)*	p-value	Cases	HR (95% CI)*	Cases	HR (95% CI)*
Age 0–39							
Control	262	1		153	1	109	1
FH	14	1.07 (0.62–1.83)	0.82	5	0.66 (0.27–1.60)	9	1.63 (0.83–3.22)
Age 40–69							
Control	1673	1		769	1	904	1
FH	66	0.73 (0.57–0.94)	0.01	32	0.79 (0.56–1.13)	34	0.69 (0.49–0.97)
Age 70+							
Control	285	1		163	1	122	1
FH	17	0.95 (0.58–1.56)	0.85	9	0.90 (0.46–1.77)	8	1.02 (0.50–2.09)

*HR from Cox proportional hazards regression stratified on matched case set. FH, familial hypercholesterolemia; HR, hazard ratio; CI, confidence interval.

Our matched analysis further showed that the HR for cancer related to poor diet, for women and men combined, was 0.82 (95% CI, 0.60–1.15) (Table 1). There were 14 incidence cases of cancer related to poor diet for women with FH and 434 for the female control population resulting in HR of 0.59 (95% CI, 0.35–1.01). The corresponding HR for men was 1.10 (95% CI, 0.71–1.69) (Supplementary Table 1). There were no difference in risk estimates between the total FH population and controls for cancer related to physical inactivity [HR 0.93 (95% CI, 0.73–1.18)], alcohol-related cancer [HR 0.98 (95% CI, 0.80–1.22)] and cancer related to body fatness and weight gain [HR 1.03 (95% CI, 0.89–1.21)] (Table 1). Neither were there any differences when the populations were stratified by sex and age groups (Supplementary Tables 2–4).

## Discussion

We found that a FH mutation was associated with 20% reduced risk of smoking-related cancer but no reduced risk of total cancer after a median of 8.7 years of follow-up. The results suggest that treatment of FH, intended to reduce the risk of CVD may also reduce the risk of cancer.

We have previously shown that cancer mortality in individuals with FH is similar to that of the general Norwegian population^13^. The current data including non-fatal cases with almost eight times more events and a matched control population supports and extends our previous findings. Importantly, the significant reduced risk of smoking-related cancer in FH is in agreement with results from the Simon Broome Register reporting reduced risk of fatal cancer [standardized mortality ratio of 0.70 (95% CI, 0.59–0.83)] mainly due to lower risk of smoking-related cancers such as respiratory and lymphatic cancer^14,15^.

Lung cancer, one of the largest contributors to smoking-related cancer, is among the most common cancer sites in Norway particularly in men^16,17^. Our results show that men with FH aged 40–69 years at baseline had a 31% lower risk of smoking-related cancer than their matched non-FH controls. The ACS consider smoking a strong risk factor for the cancer sites included in the predefined group of smoking-related cancers^11^. Hence, the reduced risk observed in the FH group is likely caused by less smoking in FH individuals than in controls. Indeed, in 2014–15, 9% of a subsample of the FH population in the present study were current smokers^18^, compared with 13% of the general population^19^ and similar differences are supported by others^20^. Furthermore, the SAFEHEART study reported that 2404 adult individuals with FH smoked less than their non-affected siblings^5^. Neil and co-workers also proposed that a reduced cancer mortality in FH might be attributable to the effects of regular medical control, including being given early and continuous lifestyle advice from early in life, resulting in better prognosis^14,15^.

Yet, in this study the incidences of cancer related to poor diet or physical inactivity were not significantly reduced in the FH population, except a tendency towards a reduced risk of cancer related to poor diet in women with FH compared with controls, with a HR of 0.59 (95% CI, 0.35–1.01). By further investigating the risk of only colorectal cancer, which accounts for the majority of cancer incidents related to poor diet in the Norwegian population, we found a HR of 0.55 (95% CI, 0.31–0.98) in FH women compared with controls (n = 12/2850 for FH and n = 400/55547 for controls, respectively). Colorectal cancer is the second most frequent cancer site in Norwegian women^16^. However, these results of post hoc analyses and small numbers should be interpreted with caution and more information on risk factors of colorectal cancer is warranted. We cannot rule out the possibility that the FH mutation per se can protect against cancer, but this is less plausible from a current biological point of view.

There was no difference in incidence of alcohol-related cancer, nor cancer related to body fatness and weight gain, between the FH population and the matched controls. In the present study, the mean age at genetic diagnosis and assumed initiation of lifestyle advice and lipid-lowering treatment in the FH cohort was 33 years with a 25-percentile of 17 years and a 75-percentile of 47 years. For those who received the FH diagnosis late in life, any pre-diagnosis unhealthy lifestyle might have already affected their cancer risk, causing any lifestyle changes to have a weaker effect on cancer risk than anticipated. On the other side, it could also be that favorable diet and lifestyle changes are not easier to comply with for individuals with FH than controls.

The relationship between statins and cancer has earlier been subject to some debate, especially in the media where negative statin-related news stories have resulted in reduced use of statins in some hypercholesterolemic individuals and accordingly increased risk of CVD^21^. A recent summary of several large randomized controlled trials (RCTs) state that there is no association between statins and the risk of cancer^22^. Yet, studies on very long-term exposure to statins are challenging to conduct. Large metaanalyses of RCTs can provide solid evidence but not for a longer exposure than the typical duration of RCTs of five years or less^23^. Development of NCDs like cancer and CHD are slow processes^24^ and data on very long time exposure are sparse. In a subsample (n = 714) of the current FH population 89% (n = 635) used statins and mainly potent statins in maximal dose. Mean duration of treatment was of 13.5 years^18^. The corresponding duration of statin treatment were mean 11.9 and 16.1 years for normal risk and very high-risk patients, respectively^18^. The median follow-up time of all individuals with FH in the present study was 12.2 years and the maximum follow-up time was 25 years (from 1992 to 2017). As statins usually are recommended from the time of FH diagnosis, this supports that the current FH population is likely to have been exposed to statins for a considerably longer time than RCTs usually lasts^23^. In secondary analysis we divided the FH population in two, those diagnosed in 1999 or earlier (n = 1104) and those diagnosed in 2000 or later (n = 4417). Compared with matched controls, HR for the FH cohort diagnosed before or equal to 1999 was 0.97 (95% CI, 0.79–1.19) and for those diagnosed in 2000 or later, the HR was 0.97 (95% CI, 0.84–1.12). Since statins were approved in 1992, the period 1992–1999 reflects possible long time use of statins compared to the period after 1999. Hence, this study could support the growing amount of evidence stating that use of statins does not increase the risk of cancer.

Strengths of the present study include the large cohort of genetically verified FH and matched controls and the long follow-up time. Since the FH mutation is inherited randomly from heterozygous parents across socioeconomic strata, there is no need to adjust for socioeconomic status. Furthermore, the use of population-based registries for collection of endpoints ensured almost no loss to follow-up. The inclusion of data from the NCoDR in addition to the CRN, allowed us to take into account deaths as competing events and hence, strengthen our results of the observed cancer risk in the FH population. The number of person years of follow-up was 71253, which is more than any other study investigating cancer risk in individuals with genetically verified FH.

Limitations include lack of information on medication use including statins, smoking prevalence and frequency, dietary habits, physical activity and other lifestyle factors, and biochemical measures such as LDL-C concentration in the study populations. There were few cancer events in FH group overall (n = 289). A greater number of FH patients or more events in the FH population are therefore likely to increase the precision of the results by reducing the wide CI observed for subgroups within the different lifestyle-related cancer sites. The FH population is likely to be more regularly followed up by physicians compared with the control population, which could cause cancer to be more easily detected in the FH population. This kind of detection bias would bias the incidence of cancer in the FH population upwards and cannot explain why we found a significantly lower risk of smoking-related cancer compared with the control population. It could, however, potentially explain why there was no difference in risk of cancer related to poor diet. Additionally, there might be limitations regarding the individuals who gives their consent to be registered in the UCCG Registry of genetically verified FH, and as only about 1/3 (n = 8511) of all individuals assumed to have FH in Norway (~25000) are diagnosed, the current sample might not reflect the total FH population in Norway^9^. The low reservation rate (2.5%) to the study however strengthens the representativeness of the results.

## Conclusion

In summary, a FH mutation and corresponding treatment was associated with reduced risk of smoking-related cancer in a FH population compared with matched controls, most likely due to a lower prevalence of smoking in the FH population. There was no difference in the risk of total cancer and cancer related to poor diet, physical inactivity, alcohol intake, and body fatness and weight gain between the FH population and the control population.

## Supplementary information

Supplementary Material

## Data Availability

Due to strict data protection rules we cannot make the materials and data freely available. However, anonymous, aggregated datasets that can replicate the results might be considered disclosed upon request.
