# Supplementary material for: Lower risk of smoking-related cancer in individuals with familial hypercholesterolemia compared with controls: a prospective matched cohort study
Source: Sci Rep. 2019 Dec 17;9:19273. doi: 10.1038/s41598-019-55682-x (PMC6917694; doi:10.1038/s41598-019-55682-x)
Supplement: Supplementary file 1 — Supplementary Material [file 41598_2019_55682_MOESM1_ESM.pdf]

Lower risk of smoking-related cancer in individuals with familial hypercholesterolemia compared with controls: a prospective matched cohort study.

Henriette W. Krogh, Msc[1*], Karianne Svendsen, PhD[1,2]*, Jannicke Igland, PhD[3], Liv J. Mundal, MD, MHA, PhD[1,2], Kirsten B. Holven, PhD[1,4] Martin P. Bogsrud, MD, PhD[4,5], Trond P. Leren, MD[5], Kjetil Retterstøl, MD, PhD[1,2]

*Contributed equally, and are both corresponding authors.

[1]Department of Nutrition, Institute of Basic Medical Sciences, University of Oslo, Norway.

[2]The Lipid Clinic, Department of Endocrinology, Morbid Obesity and Preventive Medicine, Oslo University Hospital, Norway.

[3]Department of Global Public Health and Primary Care, University of Bergen, Norway.

[4]National Advisory Unit on Familial Hypercholesterolemia, Department of Endocrinology, Morbid Obesity and Preventive Medicine, Oslo University Hospital, Norway.

[5]Unit for Cardiac and Cardiovascular Genetics, Oslo University Hospital, Norway.

Supplementary table 1: Cases of cancer related to poor diet and hazard ratios for the FH population compared with the control population, with stratification on age at baseline.

	Total study population		Women		Men	
	Cases	HR (95% CI)*	Cases	HR (95% CI)*	Cases	HR (95% CI)*
**Total study population**						
Control	801	1	434	1	367	1
FH	36	0.82 (0.60-1.15)	14	0.59 (0.35-1.01)	22	1.10 (0.71-1.69)
**Age 0-39**						
Control	101	1	54	1	47	1
FH	4	0.79 (0.30-2.14)	0	-	4	1.72 (0.62-4.78)
**Age 40-69**						
Control	577	1	299	1	278	1
FH	25	0.82 (0.55-1.22)	10	0.64 (0.34-1.20)	15	1.01 (0.60-1.70)
**Age 70+**						
Control	123	1	81	1	42	1
FH	7	0.88 (0.41-1.89)	4	0.78 (0.28-2.14)	3	1.06 (0.33-3.44)

*HR from Cox proportional hazards regression stratified on matched caseset

FH, familial hypercholesterolemia; HR, hazard ratio; CI, confidence interval.

Supplementary table 2: Cases of cancer related to physical inactivity and hazard ratios for the FH population compared with the control population, with stratification on age at baseline.

	**Total study population**		**Women**		**Men**	
	Cases	HR (95% CI)*	Cases	HR (95% CI)*	Cases	HR (95% CI)*
**Total study population**						
Control	1455	1	1231	1	224	1
FH	72	0.93 (0.73-1.18)	61	0.94 (0.72-1.21)	11	0.89 (0.48-1.63)
**Age 0-39**						
Control	266	1	243	1	23	1
FH	14	1.05 (0.61-1.80)	13	1.06 (0.61-1.85)	1	0.89 (0.12-6.62)
**Age 40-69**						
Control	1034	1	867	1	167	1
FH	53	0.97 (0.74-1.28)	43	0.95 (0.70-1.29)	10	1.10 (0.58-2.09)
**Age 70+**						
Control	155	1	121	1	34	1
FH	5	0.51 (0.21-1.26)	5	0.66 (0.27-1.64)	0	-

*HR from Cox proportional hazards regression stratified on matched caseset

FH, familial hypercholesterolemia; HR, hazard ratio; CI, confidence interval.

Supplementary table 3: Cases of alcohol-related cancer and hazard ratios for the FH population compared with the control population, with stratification on age at baseline.

	Total study population		Women		Men	
	Cases	HR (95% CI)*	Cases	HR (95% CI)*	Cases	HR (95% CI)*
**Total study population**						
Control	1718	1	1239	1	479	1
FH	90	0.98 (0.80-1.22)	58	0.89 (0.68-1.16)	32	1.23 (0.86-1.76)
**Age 0-39**						
Control	295	1	239	1	56	1
FH	18	1.22 (0.76-1.96)	13	1.09 (0.62-1.90)	5	1.78 (0.71-4.44)
**Age 40-69**						
Control	1249	1	873	1	376	1
FH	60	0.91 (0.70-1.18)	39	0.85 (0.62-1.17)	21	1.04 (0.67-1.62)
**Age 70+**						
Control	174	1	127	1	47	1
FH	12	1.13 (0.62-2.03)	6	0.79 (0.35-1.80)	6	1.95 (0.83-4.59)

*HR from Cox proportional hazards regression stratified on matched caseset

FH, familial hypercholesterolemia; HR, hazard ratio; CI, confidence interval.

Supplementary table 4: Cases of cancer related to body fatness and weight gain and hazard ratios for the FH population compared with the control population, with stratification on age at baseline.

	Total study population		Women		Men	
	Cases	HR (95% CI)*	Cases	HR (95% CI)*	Cases	HR (95% CI)*
**Total study population**						
Control	3131	1	1650	1	1481	1
FH	172	1.03 (0.89-1.21)	83	0.95 (0.76-1.19)	89	1.13 (0.91-1.40)
**Age 0-39**						
Control	400	1	301	1	99	1
FH	21	1.06 (0.68-1.64)	13	0.87 (0.50-1.51)	8	1.64 (0.80-3.38)
**Age 40-69**						
Control	2395	1	1174	1	1221	1
FH	131	1.04 (0.87-1.24)	63	1.02 (0.79-1.32)	68	1.05 (0.82-1.35)
**Age 70+**						
Control	336	1	175	1	161	1
FH	20	0.99 (0.63-1.56)	7	0.66 (0.31-1.41)	13	1.36 (0.77-2.41)

*HR from Cox proportional hazards regression stratified on matched caseset

FH, familial hypercholesterolemia; HR, hazard ratio; CI, confidence interval.
